# Development and Validation of the Minnesota Assessment of Pharmacogenomic Literacy (MAPL)

**DOI:** 10.3390/jpm12091398

**Published:** 2022-08-29

**Authors:** Josiah D. Allen, Lusi Zhang, Alyssa N. K. Johnson, Pamala A. Jacobson, Catherine A. McCarty, Amy L. Pittenger, Jeffrey R. Bishop

**Affiliations:** 1 Department of Precision Medicine and Genomic Health, St. Elizabeth Healthcare, Edgewood, KY 41017, USA; 2 Department of Experimental and Clinical Pharmacology, College of Pharmacy, University of Minnesota, Minneapolis, MN 55455, USA; 3 Department of Family Medicine and Biobehavioral Health, Medical School, University of Minnesota, Duluth, MN 55812, USA; 4 Department of Pharmaceutical Care and Health Systems, College of Pharmacy, University of Minnesota, Minneapolis, MN 55455, USA; 5 Department of Psychiatry and Behavioral Sciences, Medical School, University of Minnesota, Minneapolis, MN 55455, USA

**Keywords:** pharmacogenomics, genomic literacy, genetic counseling, psychometric validation, literacy assessment

## Abstract

Ensuring that patients have an adequate understanding of pharmacogenomic (PGx) test results is a critical component of implementing precision medicine into clinical care. However, no PGx-specific validated literacy assessment has yet been developed. To address this need, we developed and validated the Minnesota Assessment of Pharmacogenomic Literacy (MAPL^TM^). Foundational work included a scoping review of patient and general public attitudes and experiences with pharmacogenomic testing, three focus groups, readability assessments, and review by experts and members of the general public. This resulted in a 15-item assessment designed to assess knowledge in four domains: underlying concepts, limitations, benefits, and privacy. For validation, 646 participants completed the MAPL as a part of a larger survey about pharmacogenomic research and statewide PGx implementation. Two items were deemed to be “too easy” and dropped. The remaining 13 items were retained in the final MAPL with good internal reliability (Cronbach’s alpha = 0.75). Confirmatory factor analysis validated the four-domain construct of MAPL and suggested good model performance and high internal validity. The estimated coefficient loadings across 13 questions on the corresponding domains are all positive and statistically significant (*p* < 0.05). The MAPL covers multiple knowledge domains of specific relevance to PGx and is a useful tool for clinical and research settings where quantitative assessment of PGx literacy is of value.

## 1. Introduction

Pharmacogenomic (PGx) testing is increasingly entering mainstream clinical practice and is of great interest to patients and providers [1]. Ensuring that patients have an adequate understanding of PGx test results is an important component of clinical implementation and related research [2]. However, studies examining components of PGx literacy in patients have consistently identified areas of potential confusion or concern [3]. Research in disease risk genomics indicates that individuals with greater genomic literacy are better equipped to make informed decisions about obtaining genetic testing, understanding the results, and taking appropriate action based upon the findings [4,5]. 

Several validated assessments have been developed to assess patient knowledge of key genetic concepts [6,7,8,9]. These typically include basic genetic concepts, including the biological basis of heredity, disease risk genetics, as well as personal and familial considerations related to genetic testing. Unfortunately, however, these instruments tend to be focused on disease risk genomics and do not assess concepts related to genetic factors related to medication response. Within the PGx literature, some studies have used various PGx knowledge assessments to evaluate the efficacy of educational interventions, but these have been universally targeted toward healthcare providers rather than patients or the general public [10,11,12,13,14,15,16,17,18,19]. Moreover, these assessments were all developed independently by the investigators and therefore not standardized across studies. This approach presents several challenges, namely: (1) unknown reliability of the assessments being used, (2) inability to compare results across studies, and (3) unknown confounding effect of PGx literacy on clinical outcomes in studies recruiting and enrolling patients. In contrast, a standardized validated assessment tool allows for consistency across studies and assurance of the tool’s reliability and could have several uses in research (e.g., pre/post evaluation of the impact of an intervention on literacy or evaluation of PGx literacy as a potential confounder in clinical outcomes trials). In a clinical context, such a tool might be a useful screener to help assess a patient’s baseline understanding of PGx and allow the healthcare provider to target counseling to a patient’s specific misconceptions or knowledge gaps that are identified by the tool. To our knowledge, no PGx-specific, validated literacy assessment has been developed. To address this need, we developed and validated the Minnesota Assessment of Pharmacogenomic Literacy (MAPL^TM^), the results of which are presented herein.

## 2. Materials and Methods

### 2.1. Development of the MAPL

Following an established mixed-methods approach, this project utilized a multistage design with both qualitative and quantitative phases, intentional integration of findings and interpretation, resulting in a greater understanding and confidence in the conclusions [20].

In the first phase of the MAPL development process, we conducted a scoping review of qualitative research that included focus groups, semi-structured interviews (SSIs), and surveys on patient attitudes and experiences regarding PGx testing [3]. Eligible papers were analyzed qualitatively for common themes. Detailed methodology and results were previously published [3]. Briefly, from the scoping review, five primary themes emerged: (1) reasons for testing/perceived benefit, (2) patient understanding of results, (3) psychological responses to results, (4) effect on patient/provider relationship, and (5) concerns about testing/perceived harm). 

To validate these themes, particularly as they related to PGx knowledge, we conducted three in-person focus groups with patients and members of the general public. Focus groups were conducted in February and March of 2020, prior to shutdowns related to the COVID-19 pandemic. Groups 1 and 3 were recruited from a mental health support group in southeastern Minnesota, while Group 2 was recruited from an informational public session (“Mini Medical School”) on personalized medicine that was sponsored by the University of Minnesota Medical School in Minneapolis, MN. The focus group protocol was reviewed by the University of Minnesota Institutional Review Board and deemed to be exempt. Each focus group session was divided into two parts. In Part 1, the SSI and probes were developed based on the themes identified in the scoping review: benefits of testing (linked to theme 1), understanding of genetics and PGx concepts (linked to theme 2), concerns about/limitations of testing (linked to theme 5), and factors (positive or negative) that influenced one’s decision to undergo PGx testing (all themes). Because the scope of the focus groups was focused on PGx knowledge, themes 3 (psychological response to testing) and 4 (effect on patient/provider relationship) were not directly queried as a part of the SSI as they are dependent on clinical context and direct experience of a patient with PGx testing. Although not directly assessed, some reactions related to themes 3 and 4 were brought up organically by participants as a part of Part 2. In Part 2, participants were given de-identified examples of several commercially available PGx test results and, as a means of evaluating applied PGx knowledge, were queried about their understanding of various sections of the report. Sessions were recorded and transcripts were analyzed deductively for the presence/absence of previously identified themes/subthemes, then inductively for the presence of any new themes/subthemes.

With this background, we then proceeded to develop assessment items for potential inclusion in the MAPL. The items were purposefully anchored to four PGx knowledge domains based on those identified in the scoping review and tested in the focus groups: benefits of PGx testing (e.g., “Pharmacogenomic test results may tell you that a medication is likely to cause side effects”, linked to scoping review theme 1), underlying concepts (e.g., “Genes are made of DNA”, linked to scoping review theme 2), limitations of PGx testing (e.g., “Pharmacogenomic testing will help determine your diagnosis”, linked to scoping review theme 5), and privacy (e.g., “Pharmacogenomic testing companies have the right to use your data however they want without your consent”, a subset of scoping review theme 5). As with the focus groups, themes 3 and 4 were determined to be of lesser relevance to PGx knowledge assessment and potentially dependent on specific illness or provider factors and were therefore not directly translated into true/false items. 

Initially, 18 true/false items were developed. Readability was evaluated by entering the MAPL into an online readability assessment tool [21] that graded the tool using the Flesch–Kincaid Grade Level, the Gunning Fog Index, and the Flesch Reading Ease. Understandability and non-ambiguity were assessed in an online convenience sample where individuals (*n* = 7) were invited to take the assessment and provide feedback on each item. Finally, the assessment was reviewed for understandability, non-ambiguity, and scientific content by a group of experts with specialties in PGx and survey research. Ultimately, 15 items were selected for inclusion in the MAPL (Table 1). 

To quantify and test performance characteristics, we administered the MAPL to a large community sample from both metro and rural areas of Minnesota as part of a larger survey (“How Do Your Genes Fit?”) that was designed to assess interest in PGx testing and research. This research took place via the University of Minnesota Driven to Discover community research program at the Minnesota State Fair in August/September 2021 [22]. Eligibility criteria included age ≥ 18, residency in Minnesota, ability to speak English, and ability to provide informed consent. All questionnaires were entered directly into a REDCap database via iPad. In addition to the MAPL, demographics including self-reported gender, age, self-reported race and ethnicity, and educational background were collected, along with health-related characteristics including having a primary care provider, having health insurance, and number of prescription and non-prescription medications in the last 30 days. The survey also included questions related to statewide PGx initiatives and a health literacy scale, the All Aspects of Health Literacy Scale (AAHLS) [23]. Each item of functional, communicative, and critical health literacy was rated on a three-point Likert scale (0, 1, 2) and the score across these 10 questions were summed, resulting in an AAHLS total score, ranging from 0 to 20 (See Supplemental Table S1 for details on AAHLS scoring). 

The survey and study were reviewed and approved by the University of Minnesota Institutional Review Board.

### 2.2. Data Analysis

Data cleaning and analyses were performed using R Statistical Software (version 4.0.2). 

#### 2.2.1. Data Cleaning

**Figure 1:**
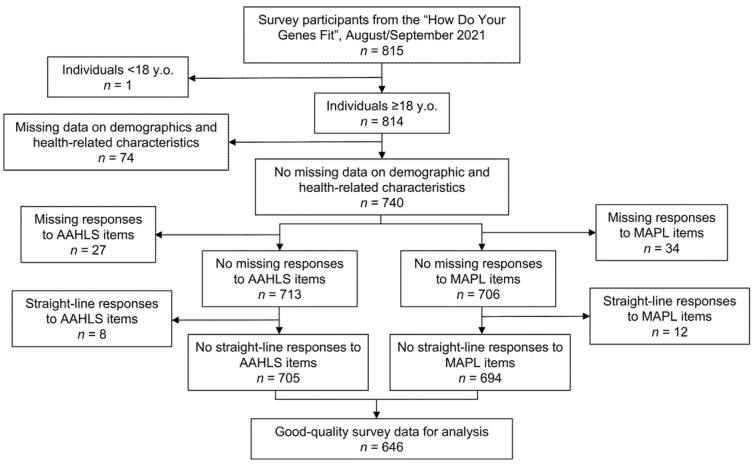
Study flowchart.

Individuals under 18 or those who did not fully complete selected sociodemographic or health-related questions were removed from the analysis. Those with missing responses to the AAHLS or MAPL were also excluded, as well as those who provided straight-line responses (e.g., all “True”, all “I don’t know”, or all “False” responses) to either the AAHLS or MAPL. The detailed inclusion and exclusion criteria and data cleaning procedure are summarized in Figure 1. 

#### 2.2.2. Statistical Analysis

Descriptive statistics summarized participants’ sociodemographic and health-related characteristics. The proportion of participants who provided correct answers to each MAPL item was calculated to assess the difficulty level. Items with >95% correct or incorrect response rates were deemed to be “too easy” or “too hard”, and the inclusion of these items into the overall MAPL were reevaluated. To calculate a MAPL total score, the response to each item was recategorized on a binary scale (0/1). The correct response was scored as 1, whereas incorrect and “I don’t know” responses were scored as 0. The scores on all included items were summed as a composite measure for which a higher scale indicated a higher PGx literacy. 

#### 2.2.3. Assessment of Internal Reliability and Construct Validity

Internal reliability of the MAPL was examined by calculating Cronbach’s alpha coefficients. An alpha value of 0.70 or higher is indicative of good reliability [24]. Confirmatory factor analysis (CFA) was performed with the “lavaan” package in R to evaluate the construct validity of the MAPL [25]. A four-factor CFA model was fitted based on the prespecified four knowledge domains in MAPL. A one-factor CFA model with one latent factor of overall PGx literacy was also examined in comparison with the validity of the four-factor construct. The model fit was evaluated using the comparative fit index (CFI; good fit if >0.90), Tucker–Lewis index (TLI; good fit if >0.90) [26], and root mean square error of approximation (RMSEA; good fit if <0.05) [27]. 

## 3. Results

### 3.1. MAPL Development

Our scoping review identified 37 relevant articles. After qualitative analysis, five primary themes were identified: (1) reasons for testing/perceived benefit, (2) patient understanding of results, (3) psychological responses to results, (4) effect on patient/provider relationship, and (5) concerns about testing/perceived harm. Within these five themes, 22 subthemes were further delineated. These results are published separately and in detail [3].

Focus groups were 57% female and enrolled a diverse range of ages and educational backgrounds (detailed demographic information can be found in Supplemental Table S2). Participants largely reported never having received genetic testing through a healthcare provider (75% no, 20% via a direct-to-consumer genetic testing company, 5% yes). Most participants reported having never received PGx testing (81% no, 19% yes). All five themes were present in all three focus groups (Supplemental Table S3). Two subthemes identified in our review of existing literature were not identified in our focus groups. These included the benefits of explaining past drug failures and implications of results for family. All other subthemes were mentioned at least once. 

Readability assessments were significantly impacted by use of the term “pharmacogenomics.” Replacing this word with the abbreviation “PGx” lowered the Flesch–Kincaid Grade Level from 9.5 to 7.3, the Gunning Fog Index from 11.0 to 10.8, and increased the Flesch Reading Ease from 49.0 to 64.8, indicating sufficient readability of the items other than this multisyllabic, technical term. The term “pharmacogenomics” was deemed to be necessary and therefore retained in the final version of the assessment.

### 3.2. MAPL Validation and Performance Characteristics

The “How Do Your Genes Fit” study enrolled 815 participants. After data cleaning, 646 participants remained for MAPL validation analysis (Figure 1). Participants’ demographic and health-related characteristics are described in Table 2 and Table 3. The population was predominantly White and nearly 60% had a bachelor’s degree or higher. 

#### 3.2.1. Descriptive Statistics

**Figure 2:**
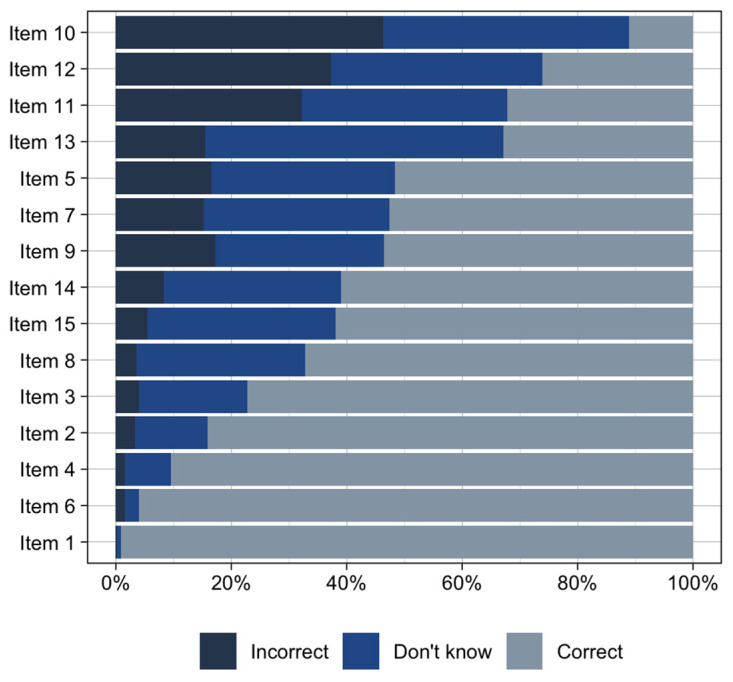
Item response distribution for 15-item MAPL.

**Figure 3:**
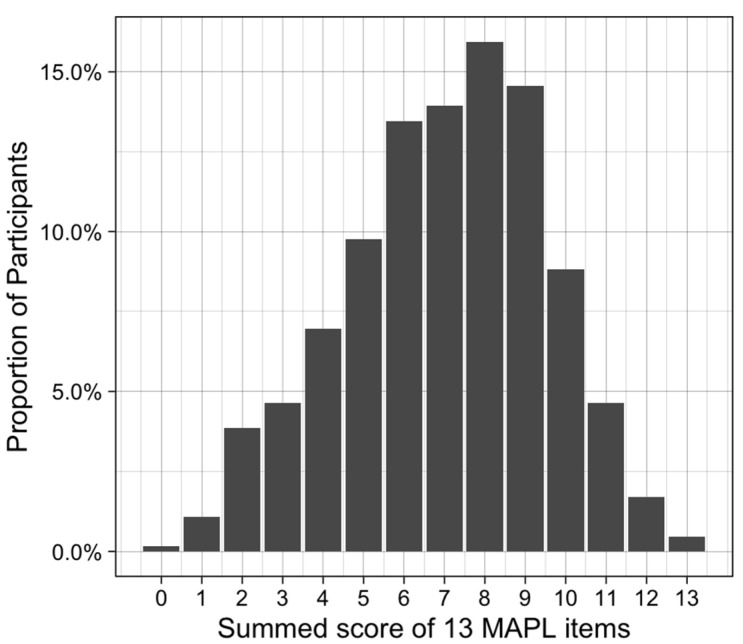
Summed score distribution for the 13-item MAPL.

**Figure 4:**
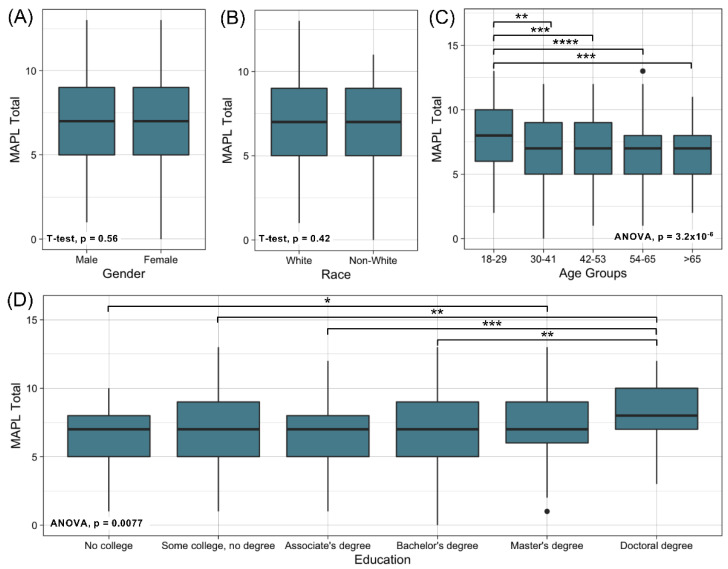
MAPL performance by demographic characteristics. (**A**). Race; (**B**). Gender; (**C**). Age; (**D**). Education. Black dots in boxplots denote individual data points that are located outside 1.5 times the interquartile range above the upper quartile and below the lower quartile. **** *p* < 0.0001; *** *p* < 0.001; ** *p* < 0.01; * *p* < 0.05.

Items 1 (“Different people will respond to medications differently”) and 6 (“Your genes are inherited from your parents”) were answered correctly by >95% of participants and were thus deemed “too easy” and removed (Figure 2). The remaining 13 items were retained in the final version of MAPL (Supplemental Table S4). The summed score of the MAPL, ranged from 0 to 13, with scores exhibiting a normal distribution (Figure 3). The mean and median correct responses were both 7. Individuals with doctoral degrees and those in the 18–29 age group scored slightly higher on the MAPL than other groups (Figure 4). There were no statistically significant differences in MAPL score by self-reported gender, self-reported ethnicity, or self-reported race, nor were there differences by health characteristics (i.e., primary care provider, health insurance, medication use). Additionally, we did not identify a significant correlation between MAPL performance and AAHLS score (Supplementary Figure S1).

#### 3.2.2. Assessment of Internal Reliability and Construct Validity

**Figure 5:**
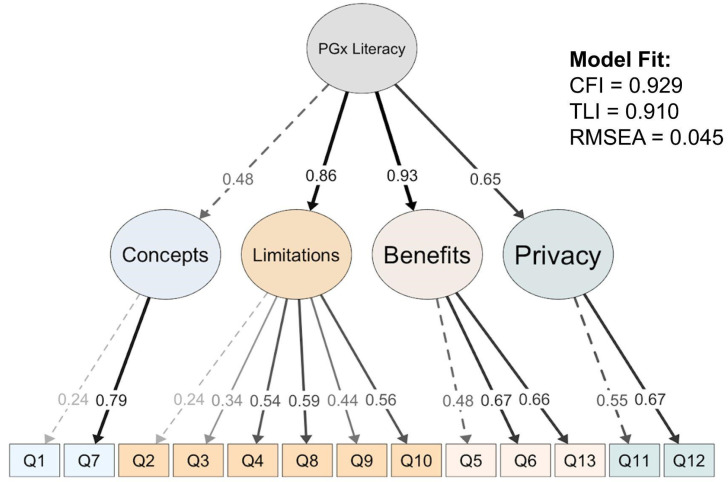
Confirmatory factor analysis (CFA) using structural equation model for the 13-item MAPL.

The remaining 13 items were retained in the final MAPL with good internal reliability (Cronbach’s alpha = 0.75). CFA validated the four-domain construct of MAPL (Figure 5) and suggested good model performance and high internal validity (CFI = 0.93, TLI = 0.91; RMSEA = 0.045). The estimated coefficient loadings across the 13 questions on the corresponding domains were all positive and statistically significant (*p* < 0.05). All four domains (Concepts, Limitations, Benefits, and Privacy) had significant positive factor loadings on a higher-level latent factor, which further corroborated the four-domain structure under the global PGx literacy. In comparison, the performance of a single-factor model of MAPL (CFI = 0.85, TLI = 0.83, RMSEA = 0.063; Supplementary Figure S2) did not outperform the four-factor model. 

## 4. Discussion

We present herein the development process and subsequent psychometric characterization of the MAPL, which is, to our knowledge, the first validated PGx-specific literacy assessment. We used a rigorous development process that involved systematic review of existing literature, patient/general public focus groups, and incorporated the input of experts and the general public on the development of specific assessment items. The MAPL demonstrated a high degree of internal reliability, and confirmatory factor analysis validated the reliability of our prespecified four-domain model. This assessment represents a tool that can be used to objectively quantify core elements of PGx literacy or identify areas for focus during patient education sessions.

Patient education and knowledge of PGx is an essential component of the clinical implementation of this element of precision medicine. Previously published efforts to increase PGx literacy include one-on-one educational interventions occurring in the clinic setting [2], education of current practitioners within health systems [13,28], and clinician education in medical and pharmacy curricula [29,30,31,32,33]. Additionally, several recent studies have characterized patient factors related to PGx literacy [34,35] and communication preferences regarding PGx concepts [36,37,38,39,40]. However, we are unaware of any additional studies that have specifically measured PGx literacy among patients or the general public. The effects of differential PGx literacy on clinical outcomes among study subjects is unknown and could be an important confounder in research studies. Moreover, our results indicate that other measures that might be used as a proxy for PGx literacy may not correlate well with actual PGx understanding. Neither health literacy (as measured by the AAHLS) nor educational attainment (except for doctoral degree attainment) were associated with MAPL performance. This suggests that, because PGx is a relatively novel concept for many, subject knowledge is not dependent on baseline health literacy or educational attainment, except for highly trained professionals at the highest levels of the educational spectrum. The MAPL provides an objective, validated method for directly assessing PGx literacy. 

Gaps in knowledge and education about PGx for patients and providers are consistently cited as a major barrier to the implementation of PGx testing in clinical workflows [41,42,43,44,45,46]. At the same time, patient interest in PGx testing remains quite high [1]. For example, in a survey conducted by Mai et al., 84% of respondents indicated a willingness to have genetic testing performed [47]. This gap between patients’ enthusiasm for PGx testing and variable understanding of important concepts underscores the need for the development of tools that help clinicians or researchers assess patient understanding. For example, 88% of respondents in Haga, et al. “reported that they understand how genetic testing can be used in healthcare very well or somewhat well” [48], but, when presented with results, almost half (45%) of participants in Olson et al. were “a little” or “not at all” confident in their ability to explain their PGx results to a friend or family member [49]. Similarly, Kastrinos et al., surveying 598 psychiatric patients, found that patients had a strong interest in PGx testing despite having low familiarity with the details [50]. This suggests that a patient’s enthusiasm and self-reported understanding of PGx may not be good predictors of the actual understanding needed to make an informed choice about testing and adequately comprehend the results and their implications. For example, the two items with the highest number of incorrect responses (Items 10 and 12) represent two common misconceptions about PGx: that PGx will identify the best medication to treat a condition and that PGx will help to identify a patient’s diagnosis. Patients who undergo PGx testing while holding these misconceptions are likely at greater risk of misinterpreting the results of testing and/or experiencing feelings of disappointment when the testing does not identify the “best” medication for them. Clinicians engaged in PGx implementation could use the MAPL as a screening tool to help focus their pre-test and post-test education on specific misconceptions identified by the MAPL. Researchers may find value in quantifying study participant knowledge of PGx in intervention or implementation studies in relation to study outcomes, or perhaps measuring change in PGx understanding as an outcome measure.

Strengths of this study include a robust development process and a large validation sample. Limitations must also be considered. The first is that the study sample used to evaluate performance characteristics was of largely European descent and had an above average level of educational attainment. These results may not be generalizable to individuals with different sociodemographic characteristics or those from specific clinical or research populations. Additional research is needed to evaluate the performance of the MAPL in other sociocultural populations, as well as among clinicians and healthcare trainees.

## 5. Conclusions

The MAPL assesses multiple knowledge domains with relevance to PGx. As prior research has demonstrated and our pre-validation work confirmed, many patients and members of the general public hold misconceptions about what PGx is and how it may be used in clinical care. We used a rigorous approach to development and psychometric validation of the MAPL. Performance scores on the MAPL exhibited a normal distribution with a reasonable dispersion of correct and incorrect answers across items. The assessment demonstrated good internal reliability in a representative sample from the general public and confirmatory factor analysis validated our prespecified knowledge domains. The MAPL represents a useful tool for clinical and research settings where quantitative assessment of PGx literacy is of value.

## Figures and Tables

**Table 1:** Minnesota Assessment of Pharmacogenomic Literacy with answers and domains.

Item Number	Question	Correct Answer	Domain
1 *	Different people will respond to medications differently.	True	Underlying Concepts
2	Genes are made of DNA.	True	Underlying Concepts
3	If a medication works for your family member, it will work for you too.	False	Limitations
4	Genes are one of many different things that can affect how you respond to a medication.	True	Limitations
5	Pharmacogenomic test results will tell you how you will respond to every medication.	False	Limitations
6 *	Your genes are inherited from your parents.	True	Underlying Concepts
7	Genes can affect how much medication is in your body after you take a pill.	True	Benefits
8	Pharmacogenomic test results may tell you that a medication is likely to cause side effects.	True	Benefits
9	Your body breaks down medications to get rid of them.	True	Underlying Concepts
10	Pharmacogenomic testing will tell you the best medication to treat your condition.	False	Limitations
11	When deciding what medication is best for you, your genetic makeup is more important than age, weight, or other medications you are taking.	False	Limitations
12	Pharmacogenomic testing will help determine your diagnosis.	False	Limitations
13	Health insurance companies can use your pharmacogenomic test results to deny coverage.	False	Privacy
14	Pharmacogenomic testing companies have the right to use your data however they want without your consent.	False	Privacy
15	Pharmacogenomic testing can tell you that you may need a different dose of a medication.	True	Benefits

* Items removed from the final assessment due to incorrect response rate < 5%.

**Table 2:** Participant demographic characteristics.

	N (*n* = 646)	%
Gender		
Men	235	36.4%
Women	408	63.2%
Other	3	0.4%
Age		
18–29	182	28.2%
30–41	93	14.4%
42–53	125	19.3%
54–65	167	25.9%
66–77	74	11.5%
78+	5	0.8%
Race		
American Indian or Alaska Native	4	0.6%
Asian	46	7.1%
Black or African American	6	0.9%
Native Hawaiian or Pacific Islanders	2	0.3%
White	545	84.4%
Multiracial	15	2.3%
Other	13	2.0%
Unknown/Prefer not to answer	15	2.3%
Hispanic, Latino, or Spanish origin		
Yes	36	5.6%
No	601	93.0%
Unknown/Prefer not to answer	9	1.4%
Education attainment		
Less than high school	3	0.5%
High school graduate/GED/equivalent	47	7.3%
Some college, no degree	119	18.4%
Associate degree	91	14.1%
Bachelor’s degree	224	37.8%
Master’s degree	92	14.2%
Doctoral degree	50	7.7%

**Table 3:** Participant health-related characteristics.

	*n* (Mean) (*n* = 646)	% (SD)
Having primary care provider		
Yes	541	83.7%
No	105	16.3%
Having health insurance		
Yes	614	95.0%
No	32	5.0%
The number of prescription medications in the last 30 days		
0	228	35.3%
1	259	40.1%
2	113	17.5%
3+	46	7.1%
The number of non-prescription medications in the last 30 days		
0	116	18.0%
1	338	52.3%
2	128	19.8%
3+	64	9.9%
All Aspects of Health Literacy Scale (AAHLS) total score	16.4	2.7

## Data Availability

The data presented in this study are available on request from the corresponding author.

## Supplementary Materials

The following supporting information can be downloaded at: https://www.mdpi.com/article/10.3390/jpm12091398/s1, Table S1: AAHLS scoring methodology; Table S2: Focus group demographics and knowledge self-assessment; Table S3: Focus group example quotations; Figure S1: Distribution of MAPL total score in comparison with the AAHLS total score (A) and by AAHLS score tertile groups (B); Figure S2: One-factor confirmatory factor analysis (CFA) model for 13-item MAPL. Table S4: Final 13 item MAPL. Information on licensing and terms of use may be accessed at https://license.umn.edu/product/minnesota-assessment-of-pharmacogenomic-literacy-mapl (accessed on 25 August 2022).
