# Supplementary material for: Development and Validation of the Minnesota Assessment of Pharmacogenomic Literacy (MAPL)
Source: J Pers Med. 2022 Aug 29;12(9):1398. doi: 10.3390/jpm12091398 (PMC9506235; doi:10.3390/jpm12091398)
Supplement: Supplementary file 1 [file jpm-12-01398-s001.zip › jpm-1806318-supplementary.pdf]

Table S1. AAHLS scoring methodology.
In general, “rarely” responses were scored as 0 and “often” responses were scored as 2. This was reversed for FQ1 and FQ3 to match the directionality of these two items (i.e., higher score corresponding to higher literacy). The maximum score was 20.

Question	Scoring
FQ1 - How often do you need someone to help you when you are given information to read by your doctor, nurse, or pharmacist?	Rarely = 2, Sometimes = 1, Often = 0
FQ2 - When you do need help, can you easily get hold of someone to assist you?	Rarely = 0, Sometimes = 1, Often = 2
FQ3 - Do you need help to fill in official documents?	Rarely = 2, Sometimes = 1, Often = 0
CO1 - When you talk to a doctor, nurse, or pharmacist, do you give them all the information they need to help you?	Rarely = 0, Sometimes = 1, Often = 2
CO2 - When you talk to a doctor, nurse, or pharmacist do you ask the questions you need to ask?	Rarely = 0, Sometimes = 1, Often = 2
CO3 - When you talk to a doctor, nurse, or pharmacist do you make sure they explain anything that you do not understand?	Rarely = 0, Sometimes = 1, Often = 2
CR1 - Are you someone who likes to find out lots of different information about your health?	Rarely = 0, Sometimes = 1, Often = 2
CR2 - How often do you think carefully about whether health information makes sense in your particular situation?	Rarely = 0, Sometimes = 1, Often = 2
CR3 - How often do you try to work out whether information about your health can be trusted?	Rarely = 0, Sometimes = 1, Often = 2
CR4 - Are you the sort of person who might question your doctor, nurse, or pharmacist's advice based on your own research?	Yes, definitely = 2, Maybe/sometimes = 1, Not really = 0
EMP1 - Do you think that there are plenty of ways to have a say in what the government does about health?	Not scored
EMP2 - Within the last 12 months have you taken action to do something about a health issue that affects your family/community?	Not scored
EMP3 - What do you think matters most for everyone's health? (Please tick one answer only.) 1. Information and encouragement to lead healthy lifestyles. 2. Good housing, decent jobs, & good local facilities	Not scored

Table S2. Focus group demographics and knowledge self-assessment

	N (n = 21)	%
Gender		
Men	9	57%
Women	12	43%
Age*		
18-24	0	0%
25-34	2	10%
35-44	4	20%
45-54	2	10%
55-64	6	30%
65-74	4	20%
75+	2	10%
Education attainment		
Some high school or less	0	0%
High school diploma	3	14%
Some college	3	14%
2-year degree	5	24%
4-year degree	5	24%
Some graduate school	1	5%
Master's degree	2	9.5%
Doctoral degree	2	9.5%
Received genetic testing*		
No	15	75%
Yes, through a direct-to-consumer source	4	20%
Yes, through a healthcare provider	1	5%
Received pharmacogenetic testing		
No	17	81%
Yes	4	19%
Currently taking prescription medications		
Yes	18	86%
No	3	14%
How much do you feel that you know about genetics as it is used in the medical setting?		
None	5	24%
A little bit	7	33%
Some	8	38%
A lot	1	5%
How much do you feel that you know about how medications work in the body?		
None	4	19%
A little bit	6	29%
Some	11	52%
A lot	0	0%

* Missing data: age (n = 1), received genetic testing (n = 1). Percentages may not equal 100% due to rounding.

Table S3. Focus group example quotations.

Theme	Subtheme	Example quotations
Reasons for testing/ perceived benefit	Medication selection/dosing	• *"I would be wondering what depression medication will work for me. It was my number one goal for getting the testing if I can get the testing. That's what I would want to know because trying different meds, waiting, and side effects and all that. It's nice to just go straight to one."* (Participant 1.4)<br>• *"I the patient don't have to go through two years of experimentation with drugs for me to find out that they don't work well, or they cause weight gain, or all of the potential side effects."* (Participant 2.1)
	Side effect reduction	• *"Hopefully to reduce the amount of side effects with a medication that isn't appropriate. You can figure out which ones will be right so you can limit side effects."* (Participant 1.10)
	Optimization of future medications	• *"Knowing ahead of time what I might react best to, it's better for me, it's better for my doctor to know ahead of time. This report is really good."* (Participant 3.3)
	Explained past drug failures	None identified.
	Implications of results for family	None identified.
	Non-medical benefits	• *"I would be concerned about my data, but I would be okay if they could de-identify it and use it for research and help others. I'd be completely okay with that."* (Participant 1.4)
Understanding of results	General PGx knowledge	• Facilitator: *"If you were to explain how genetic testing works to a friend or to a family member, how would you describe it?"* Participant 1.10: *"It's magic."*<br>• *"Environmental factors like the people that are submitted to radiation in close to a nuclear factory or something like that, it will totally modify your DNA."* (Participant 2.2)
	Information level preference and delivery	• Facilitator: "*Would everyone want to see the informative ones even if there's not necessarily strong data behind it just yet?*" Participant 2.3: *Oh, yes, if my $300 pays for it. Get everything I can get off of it."*<br>• *"It almost seems like this is something that the provider shouldn't even hand to you. It seems like this is something for them to interpret and then tell you, not just [hand it to you and say], 'here you go.'"* (Participant 1.3)

	Terminology confusion	• *"Not being from the medical community or scientific, I don't know what a lot of the things mean. It's not like layman's terms. I have a hard time understanding what it actually means."* (Participant 3.1)<br>• *"All of this to me, would be mumbo jumbo. My best friend isn't medical savvy, this would be mumbo jumbo to her. She'd be like, "What am I reading?". They wouldn't understand it, they would have to say, "Okay."* (Participant 3.3)
	PGx testing vs. disease/trait testing	• *"To do the regular DNA test and you look back to see what you are predisposed to having, or maybe what you do have a little bit of."* (Participant 2.3)
	Uncertainty about implications of results	• *"After the testing, they throw it out to you you're a fast metabolizer, you're a slow metabolizer. I definitely need more information on what it means."* (Participant 1.5)
Psychological responses to results	Positive responses to testing	• *"I think this pharmacogenetics will allow also the physician to tailor the right treatment for the patient, like knowing all the things that he's going to need or the things that are going to work or are not going to work, because I think the physicians, sometimes they know what they have to prescribe, but they don't know, in fact, what's happening in your body."* (Participant 2.2)<br>• *"I mean, the whole purpose of this is to get to the genes of it. I would expect this to be more accurate and more in-depth and more precise than a normal CBC or UA or that type of a lab."* (Participant 3.2)
	Neutral/negative responses to testing	• *"I expected like the world and I am disappointed that it's not going to give me the world. I guess I just have really high expectations and I expect the world"* (Participant 1.6)<br>• *"I just don't know how much faith I put in any of these genetic testings, I've done the 23 in me and ancestry and there's variances between the two and knowing nothing about anything of my genetic disposition. How could two companies come up with two different things? I used the same spit."* (Participant 3.6)
	Confidence/hope in drug therapy	• *"It's not as good as I would hope it could be but that gives hope for the future as well, I think."* (Participant 1.2)
Effect on patient/	Sharing of results with providers	• *"If they found something that yes, it's going to make me better down the road. I'd want them*

provider relationship		*to share with my primary care*." (Participant 1.1)<br>• Facilitator: "*If that situation were to happen and your psychiatrist didn't pass [the PGx] information along…, would you feel that he/she didn't necessarily do his job entirely?*" All Group 1 participants: "*Yes*."
	Provider implementation of results	• *"[I would want my provider to tell me] how are the results going to be used in my health care. How will the results be filed in my health care record so that any provider that I see regardless of whether and how they'll be able to use them, and what other information can they get from the test that would be beneficial from the healthcare point."* (Participant 1.3)<br>• *"I would want to know that they're going to continue in communication and observation with me as I'm starting out whatever the recommended medications are."* (Participant 1.9)<br>• *"This report here really is a basis for conversation with the doctor, right? That's what I would expect it to be, that I wouldn't be given this and say, "Here, you go away and think about it", when there isn't a good discussion about it."* Participant 2.6)
	Confidence in providers	• *"I think my first question would be, what is your experience with the testing you've already done, has it been proved to be beneficial or is it a pie in the sky?"* (Participant 3.5)<br>• *"I don't have an issue with trusting my doctor. I know she's on my side."* (Participant 2.6)
	Data privacy/security/abuse of information	• *"I'd want to make sure that the testing was - I knew about it, the company that did it, and my health care provider, period, nobody else. Couldn't be used for-- Not that I'm hiding anything or plan on committing a crime, but I don't want it to be able to go anywhere."* (Participant 1.7)
Concerns about testing/ perceived harm	Cost of test/insurance coverage	• *"I think cost would be important for me. It's one thing to pay 300, it's another thing to pay 2,000 or something for the test. I would want to know exactly what information that test is capable of giving me, and I would like them to tell me why it would be a good idea for me to do it."* (Participant 1.8)
	Scientific/technical limitations	• *"I [would want my provider to tell me] that this is not hard science here. They're probably parts of it that are quantifiable like metabolize*

		things, but in terms of what's going to work for you, I don't know if that's real science yet." (Participant 1.5)<br>• *"I would hope that someday it reaches that point where it is that definitive because like you said it is genes but not like it's there yet. We are pretty new as far as genetic research goes, aren't we? Twenty years we've been doing this only."* (Participant 3.6)
	Insurance discrimination	• *"Eventually, we may get to the time when it's not confidential, and the litigation will blow up in our country. As well as your insurance costs, it'll be chaos… If they do, I don't know how a patient can get proper treatment. It's a real dilemma."* (Participant 2.3)
	Secondary findings	• "*The other aspect of it too is further insurance claims being denied that if they use a genetic testing and say, "Hey, this guy is predisposed to liver cancer, pancreatic cancer, we should drop them."* (Participant 1.5)

Figure S1. Distribution of MAPL total score in comparison with the AAHLS total score (A) and by AAHLS score tertile groups (B).

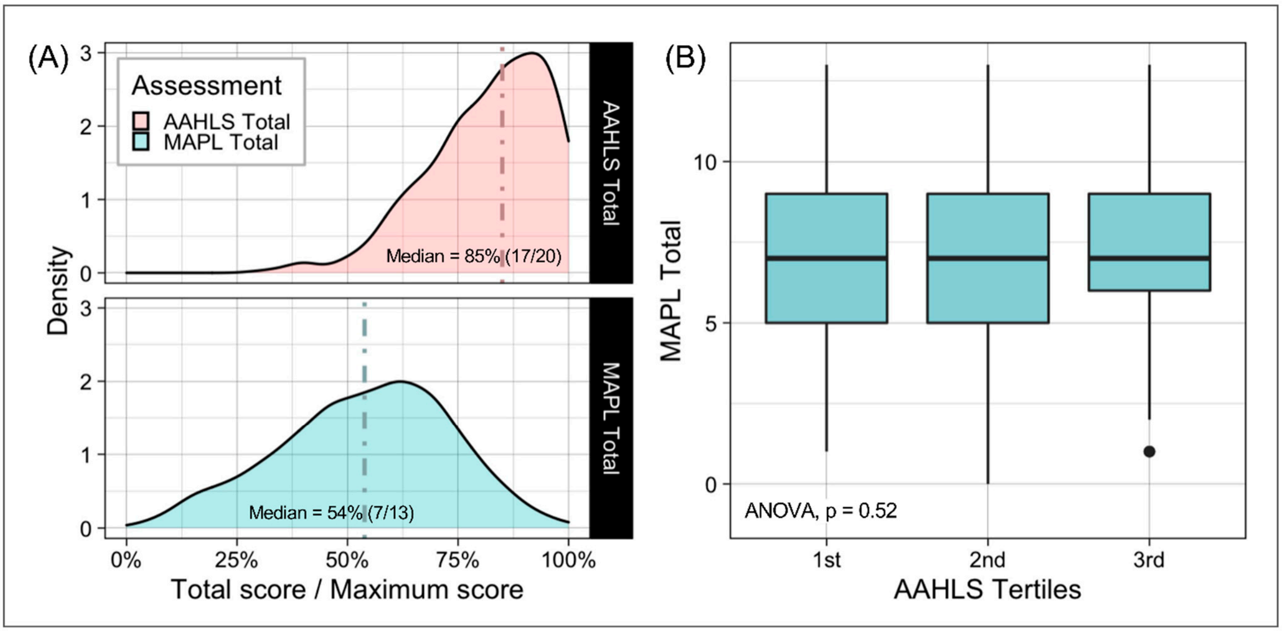

Figure S2. One-factor confirmatory factor analysis (CFA) model for 13-item MAPL.

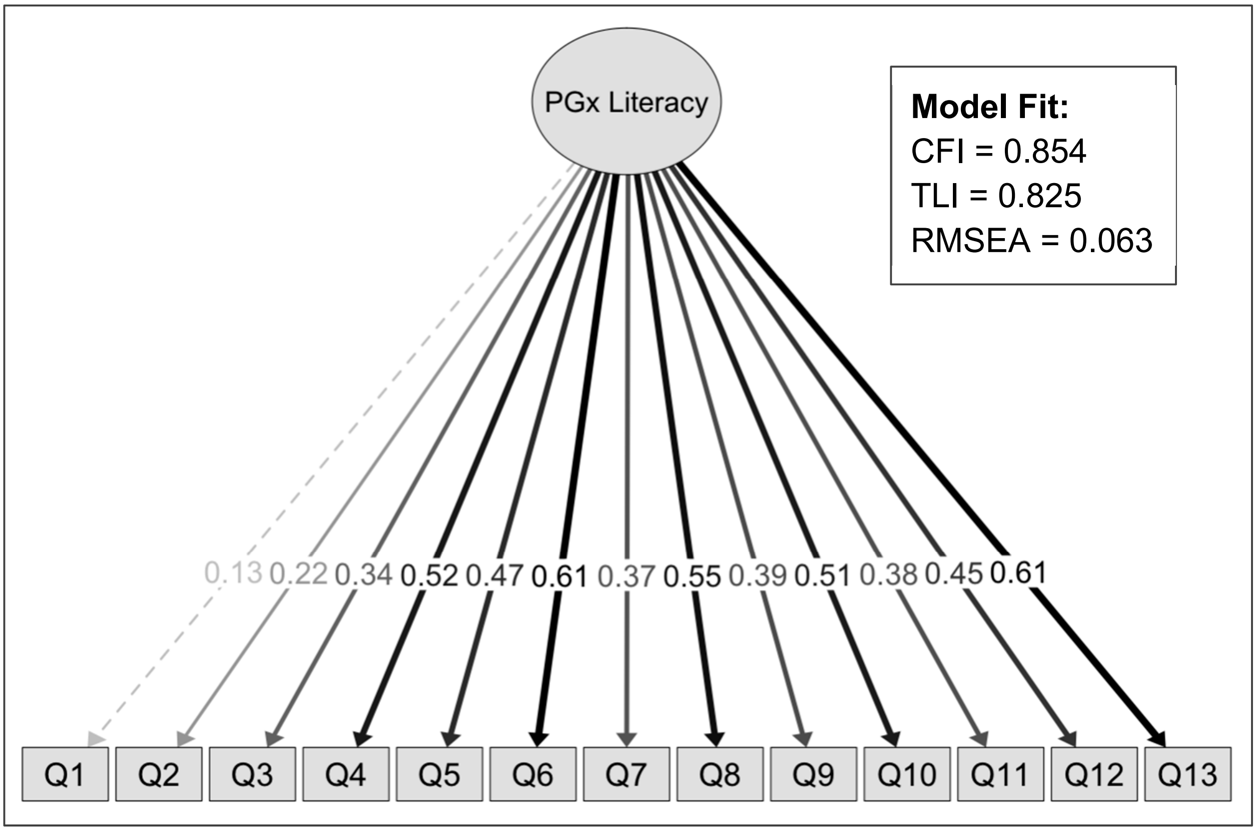

Table S4. Final 13 item MAPL

Number	Question	Answer	Domain
1	Genes are made of DNA	True	Underlying concepts
2	If a medication works for your family member, it will work for you too.	False	Limitations
3	Genes are one of many different things that can affect how you respond to a medication.	True	Limitations
4	Pharmacogenomic test results will tell you how you will respond to every medication.	False	Limitations
5	Genes can affect how much medication is in your body after you take a pill.	True	Benefits
6	Pharmacogenomic test results may tell you that a medication is likely to cause side effects.	True	Benefits
7	Your body breaks down medications to get rid of them.	True	Underlying concepts
8	Pharmacogenomic testing will tell you the best medication to treat your condition.	False	Limitations
9	When deciding what medication is best for you, your genetic makeup is more important than age, weight, or other medications you are taking.	False	Limitations
10	Pharmacogenomic testing will help determine your diagnosis.	False	Limitations
11	Health insurance companies can use your pharmacogenomic test results to deny coverage.	False	Privacy
12	Pharmacogenomic testing companies have the right to use your data however they want without your consent.	False	Privacy
13	Pharmacogenomic testing can tell you that you may need a different dose of a medication.	True	Benefits
